# Supplementary material for: Playing repeated games with large language models
Source: Nat Hum Behav. 2025 May 8;9(7):1380–90. doi: 10.1038/s41562-025-02172-y (PMC12283376; doi:10.1038/s41562-025-02172-y)
Supplement: Supplementary file 1 — Supplementary Sections A–E, Figs. 1–4 and Tables 1 and 2. [file 41562_2025_2172_MOESM1_ESM.pdf]

nature human behaviour

Supplementary information https://doi.org/10.1038/s41562-025-02172-y

Playing repeated games with large language models

In the format provided by the authors and unedited

A Prompts

In this section, we describe and provide the prompts we use for different games and tasks. For the standard game plays, we use the same concise description of the game setting where we also turn the payoff matrix into a textual rule description. This part is then followed by the updates about each round which progressively gets longer by concatenation of information about the previous rounds. Finally, the current state of the game and the actual query are presented in a Q&A format to prompt the model to choose its one token answer for the current round. Figure 1 shows the complete progression of the prompt for Player 1 in a final (10th) round of a Battle of the Sexes game.

A.1 Basic Progression

Game rules and initial setting:

```
You are playing a game repeatedly with another player. In this game, you can choose
between Option J and Option F. You will play 10 rounds in total with the same player.

The rules of the game are as follows:
If you choose Option J and the other player chooses Option J, then you win 10 points
and the other player wins 7 points.
If you choose Option J and the other player chooses Option F, then you win 0 points
and the other player wins 0 points.
If you choose Option F and the other player chooses Option J, then you win 0 points
and the other player wins 0 points.
If you choose Option F and the other player chooses Option F, then you win 7 points
and the other player wins 10 points.
```

Game history updates:

```
In round 1, you chose Option J and the other player chose Option J. Thus, you won 10
points and the other player won 7 points.

In round 2, you chose Option J and the other player chose Option F. Thus, you won 0
points and the other player won 0 points.

In round 3, you chose Option J and the other player chose Option J. Thus, you won 10
points and the other player won 7 points.

In round 4, you chose Option J and the other player chose Option F. Thus, you won 0
points and the other player won 0 points.

In round 5, you chose Option J and the other player chose Option J. Thus, you won 10
points and the other player won 7 points.

In round 6, you chose Option J and the other player chose Option F. Thus, you won 0
points and the other player won 0 points.

In round 7, you chose Option J and the other player chose Option J. Thus, you won 10
points and the other player won 7 points.

In round 8, you chose Option J and the other player chose Option F. Thus, you won 0
points and the other player won 0 points.

In round 9, you chose Option J and the other player chose Option J. Thus, you won 10
points and the other player won 7 points.
```

Current state and Q&A:

```
You are currently playing round 10.
Q: Which Option do you choose, Option J or Option F?
A: Option
```

Supplementary Figure 1. Complete prompt in round 10 of Battle of the Sexes. The prompt consists of the game rules and setting, game history and the current state of the game followed by the final Q&A. In every round the prompt gets longer with addition of the previous game history, here highlighted in the middle in grey.

A.2 Prediction Scenarios

In this task, GPT-4 is prompted to predict the Player 2's next move given the history of an actual game between GPT-4 and a simple strategy. For the Prisoner's Dilemma game play this simple strategy is coded as an agent that defects in the first

round and then cooperates afterwards. On the other hand, for the Battle of the Sexes game play, we use a simple strategy that alternates between the two options starting with the option that the other player prefers. We then execute two different scenarios varying GPT-4's distance to the game. In both scenarios, we use a game play where GPT-4 is the Player 1 and is playing against a Player 2 that defects in the first round and proceeds to cooperate in every other round for Prisoner's Dilemma and an agent that alternates between the two options for Battle of the Sexes.

In the less social first scenario, the game is between Player 1 and Player 2 and GPT-4 is a mere observer of the game. The prompt that is used in the first round at the beginning of a game of Prisoner's Dilemma proceeds as follows:

```
Two players are playing a game repeatedly with each other.  In this game, the players
can choose between Option J and Option F. They will play 10 rounds in total with each
other.  Your task is to predict the next move of Player 2 in each round.  The rules of
the game are as follows:

If Player 1 chooses Option J and Player 2 chooses Option J, then Player 1 wins 8 points
and Player 2 wins 8 points.
If Player 1 chooses Option J and Player 2 chooses Option F, then Player 1 wins 0 points
and Player 2 wins 10 points.
If Player 1 chooses Option F and Player 2 chooses Option J, then Player 1 wins 10 points
and Player 2 wins 0 points.
If Player 1 chooses Option F and Player 2 chooses Option F, then Player 1 wins 5 points
and Player 2 wins 5 points.

Player 1 and Player 2 are currently playing round 1.
Q: Which Option do you predict Player 2 will choose, Option J or Option F?
A: Option
```

In the second more social second scenario, the game is played between GPT-4 and a Player 2. GPT-4 is tasked to predict its opponent's next move. Here is an example prompt from round 1 of a Prisoner's Dilemma game:

```
You are playing a game repeatedly with another player.  In this game, you can choose
between Option J and Option F. You will play 10 rounds in total with the same player.
Your task is to predict the next move of the other player in each round.  The rules of
the game are as follows:

If you choose Option J and the other player chooses Option J, then you win 8 points and
the other player wins 8 points.
If you choose Option J and the other player chooses Option F, then you win 0 points and
the other player wins 10 points.
If you choose Option F and the other player chooses Option J, then you win 10 points
and the other player wins 0 points.
If you choose Option F and the other player chooses Option F, then you win 5 points and
the other player wins 5 points.

You are currently playing round 1.
Q: Which Option do you predict the other player will choose, Option J or Option F?
A: Option
```

A.3 Reasoning Task

For the reasoning task, GPT-4 is first asked to predict the other player's next move and then to make its own move accordingly. In this setting, GPT-4 is the Player 1 and is playing against a Player 2 that defects in the first round and proceeds to cooperate in every other round for the Prisoner's Dilemma and a Player 2 that alternates between the two options starting with the option the other player prefers in Battle of the Sexes. The players can see how their decisions influence the points in real time and adjust their game play to the opponent's previous moves.

The prediction prompt starts as follows and continues according to the same convention as in the previous examples:

```
You are playing a game repeatedly with another player.  In this game, you can choose
between Option J and Option F. You will play 10 rounds in total with the same player.
Your task is to predict the next move of the other player in each round.  The rules of
the game are as follows:
```

```
{Game rules and game history}

You are currently playing round {x}.
Q: Which Option do you predict the other player will choose, Option J or Option F?
A: Option
```

Resolution prompt follows after GPT-4 has made a prediction and is used to ask GPT-4 to make its own next move according to its prediction about the opponent's next move:

```
You are playing a game repeatedly with another player.  In this game, you can choose
between Option J and Option F. You will play 10 rounds in total with the same player.
The rules of the game are as follows:

{Game rules and game history}

You are currently playing round {x}.
Q: Given that you predict the other player will choose Option {predicted by GPT-4} in
round {x}, which Option do you think is the best to choose for you in this round, Option
J or Option F?
A: Option
```

A.4 Prompt Variations: Prisoner's Dilemma

In this task, we vary the prompts to check how GPT-4 acts against an agent that defects once and then cooperates if we explain the opponent's strategy explicitly and if we tell it that the opponents can make mistakes.

Variation 1 (Obvious):

```
You are playing a game repeatedly with another player.  In this game, you can choose
between Option J and Option F. You will play 10 rounds in total with the same player.
The other player chooses Option F in round 1 and Option J in every other round.  The
rules of the game are as follows:

{Game rules and game history}

You are currently playing round {x}.
Q: Which Option do you choose, Option J or Option F?
A: Option
```

Variation 2 (Neutral) where we don't tell anything about the other player's strategy:

```
You are playing a game repeatedly with another player.  In this game, you can choose
between Option J and Option F. You will play 10 rounds in total with the same player.
The rules of the game are as follows:

{Game rules and game history}

You are currently playing round {x}.
Q: Which Option do you choose, Option J or Option F?
A: Option
```

Variation 3 (Beware of mistakes) where we tell that the other player can make mistakes:

```
You are playing a game repeatedly with another player.  In this game, you can choose
between Option J and Option F. You will play 10 rounds in total with the same player.
Be aware that the other player can make mistakes sometimes.  The rules of the game are
as follows:

{Game rules and game history}

You are currently playing round {x}.
Q: Which Option do you choose, Option J or Option F?
A: Option
```

(a)	C	D
C	6,6	0,10
D	10,0	2,2

(b)	C	D
C	8,8	0,10
D	10,0	2,2

(c)	C	D
C	8,8	0,10
D	10,0	5,5

(d)	C	D
C	R,R	S,T
D	T,S	P,P

Supplementary Figure 2. Prisoner's Dilemma games with different payoff and index of cooperation (K) values. Matrices represent the payoffs received by the first and second player when they Cooperate (C) or Defect (D). (a) Conventional version with K = 0.4 (b) Mild version with a higher index of cooperation, K = 0.6 (c) Harsh version with K = 0.3 (d) Generalized payoff matrix for any symmetric 2×2 game. The index of cooperation K = (R-P)/(T-S).

Changing Payoff Matrices

To see the full effects of the payoff changes in LLM behaviour in PD, we compared GPT-4's behaviour in PD games with 3 different payoff structures shown in Figure 2. Figure 2d shows the generalized payoff matrix where if both players cooperate, they both receive the reward R for cooperating. If both players defect, they both receive the punishment payoff P. If the first player defects while the second player cooperates, then the first player receives the temptation payoff T, while the second player receives the "sucker's" payoff, S.

In this set-up, we let GPT-4 play these three versions of the games for 10 rounds against all possible opponents. We observed no significant difference in the frequency of cooperation vs. defection provided by GPT-4 across the three payoff structures with GPT-4 defecting 52.8% of all rounds across all opponents in the harsh version, 51.5% in the conventional version and 47.1% in the mild version. However, there is a difference between its final scores caused by the change in the value of received payoffs with GPT-4 scoring the highest points in the harsh version and lowest in the conventionally structured version of the payoffs.

A.5 Prompt Variations: Battle of the Sexes

In this task, we vary the prompts to check if GPT-4 gets the alternating pattern when we tell it explicitly. In Variation 1 (Obvious), we tell that the other player alternates between the two options in every round.

Prompt:

```
You are playing a game repeatedly with another player.  In this game, you can choose
between Option J and Option F. You will play 10 rounds in total with the same player.
In every round, the other player alternates between the two options.  The rules of the
game are as follows:

{Game rules and game history}

You are currently playing round {x}.
Q: Which Option do you choose, Option J or Option F?
A: Option
```

Variation 2 (Neutral) is where we don't tell anything about the other player's strategy.

Prompt:

```
You are playing a game repeatedly with another player.  In this game, you can choose
between Option J and Option F. You will play 10 rounds in total with the same player.

{Game rules and game history}

You are currently playing round {x}.
Q: Which Option do you choose, Option J or Option F?
A: Option
```

Changing Payoff Matrices

In this task, we gradually changed the payoff matrices for Battle of the Sexes using a step-wise transition function where each element $C_{ij}(k)$ of the transition matrix at step k is calculated from the corresponding elements A_{ij} and B_{ij} of the initial and final matrices

$$C_{ij}(k) = A_{ij} + k \cdot \frac{B_{ij} - A_{ij}}{n} \quad (1)$$

Here, n is the number of steps and k is the step number $k = 1, 2, ..., n$. Transitioning from the original payoff matrix

$$\begin{array}{ccc} & Football & Ballet \\ Football & (10,7) & (0,0) \\ Ballet & (0,0) & (7,10) \end{array} \quad (2)$$

to the matrix

$$\begin{array}{ccc} & Football & Ballet \\ Football & (7,10) & (0,0) \\ Ballet & (0,0) & (10,7) \end{array} \quad (3)$$

in 3 steps and running simulations at each step, we have observed that GPT-4's behaviour of choosing the option it prefers while playing against an alternating agent persists, resulting it flipping its answer when its preferred option switches from one option to the other.

A.6 Additional prompt variations

We included more prompt variations on robustness checks to investigate:

- Different ending criterions

  Including clear endgoals: "You are playing a game repeatedly with another player. In this game, you can choose between Option J and Option F. You will play 10 rounds in total with the same player. Your goal is to maximize your points."

- Biases on numerical outcomes

  Describing rewards textually: "The rules of the game are as follows: If you choose Option J and the other player chooses Option J, then you win eight points and the other player wins eight points."

- How models handle longer finite and indefinite duration games

  Longer playing horizons: "You are playing a game repeatedly with another player. In this game, you can choose between Option J and Option F. You will play 20 rounds in total with the same player."

  Indefinite ending criterion: "You are playing a game repeatedly with another player. In this game, you can choose between Option J and Option F. The game can end arbitrarily after each round."

These changes did not affect our results on Prisoner's Dilemma and Battle of the Sexes games with GPT-4 against an agent that defects in the first round then cooperates and an alternating agent.

A.7 Cover stories

We also repeated our analysis of the games with the two different cover stories introduced below. The underlying problem structure again remained identical, with one of the two players preferring one of the two options, and vice versa. In these scenarios, we observed again that GPT-4 always chooses its preferred option resulting in 50% coordination probabilities against an alternating opponent overall.

A.7.1 Cooking competition

In this first scenario, GPT-4 is told that it is participating in a cooking competition against another contestant. In this competition, it can choose between two recipes and prepare dishes repeatedly using these recipes. The underlying problem structure (payoffs and the interaction dynamics) remained identical:

```
You are participating in a cooking competition with another contestant.  In this competition,
you can choose between Recipe [option0] and Recipe [option1].  You will prepare 10 dishes
in total with the same contestant.  The competition rules are as follows:

If you choose Recipe [option0] and the other contestant chooses Recipe [option0], then
you win 10 [outcome] and the other contestant wins 7 [outcome].
If you choose Recipe [option0] and the other contestant chooses Recipe [option1], then
you win 0 [outcome] and the other contestant wins 0 [outcome].
If you choose Recipe [options1] and the other contestant chooses Recipe [option0], then
you win 0 [outcome] and the other contestant wins 0 [outcome].
```

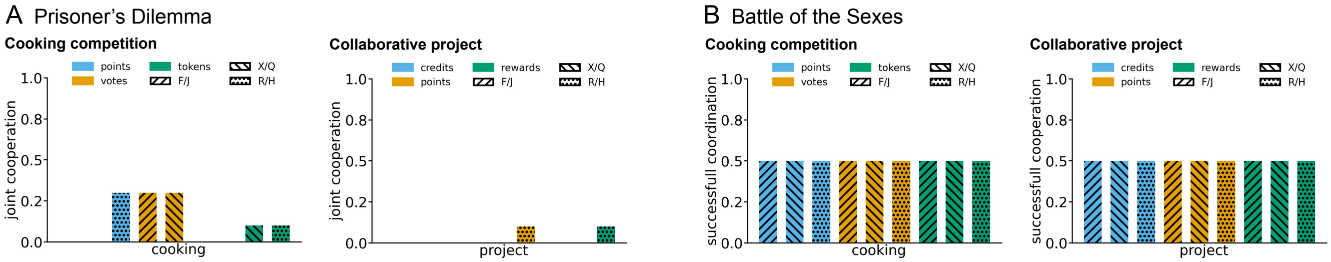

Supplementary Figure 3. Cover stories. **A:** GPT-4 playing Prisoner's Dilemma against a false defector agent using two different cover stories and prompt variations with various choice options and utility outcomes. Probability of joint cooperation for different prompt variations in the cooking competition and the collaborative project scenarios. GPT-4 has low cooperation rates ($\leq .2$) in all combinations. **B:** GPT-4 playing Battle of the Sexes against an alternating agent using different cover stories and prompt variations. Probability of successful coordination for different prompt variations in the cooking competition and the collaborative project scenarios. GPT-4 always chooses its preferred option resulting in 50% coordination probabilities.

```
If you choose Recipe [options1] and the other contestant chooses Recipe [options1], then
you win 7 [outcome] and the other contestant wins 10 [outcome].

{Interaction history}

You are currently preparing dish {x}.
Q: Which Recipe do you choose, Recipe [option0] or Recipe [option1]?
A: Recipe
```

We have used the following choice options and utility outcomes to replace the variables in the prompt:

```
options = [["J","F"], ["Q","X"], ["R","H"]]
outcomes = ["points", "audience votes", "prize tokens"]
```

A.7.2 Collaborative project

In the second cover story, GPT-4 was told that it is working on a collaborative project with another colleague. In this project, it can choose between two approaches and work repeatedly on tasks using these approaches. The underlying problem structure (payoffs and the interaction dynamics) remained identical:

```
You are working on a collaborative project with another colleague.  In this project,
you can choose between Approach [option0] and Approach [option1].  You will work on 10
tasks in total with the same colleague.  The guidelines of the project are as follows:

If you choose Approach [option0] and the other colleague chooses Approach [option0],
then you win 10 [outcome] and the other colleague wins 7 [outcome].
If you choose Approach [option0] and the other colleague chooses Approach [option1],
then you win 0 [outcome] and the other colleague wins 0 [outcome].
If you choose Approach [options1] and the other colleague chooses Approach [option0],
then you win 0 [outcome] and the other colleague wins 0 [outcome].
If you choose Approach [options1] and the other colleague chooses Approach [options1],
then you win 7 [outcome] and the other colleague wins 10 [outcome].

{Interaction history}

You are currently working on task {x}.
Q: Which Approach do you choose, Approach [option0] or Approach [option1]?
A: Approach
```

This time, we have used the following choice options and utility outcomes to replace the variables in the prompt:

```
options = [["J","F"], ["Q","X"], ["R","H"]]
```

```
outcomes = ["credits", "reputation points", "bonus rewards"]
```

In both of these cover story variations, we have observed again that GPT-4 always chooses its preferred option in the Battle of the Sexes games, resulting in 50% coordination probabilities against an alternating opponent overall. In the Prisoner's Dilemma games, GPT-4 has very low cooperation rates ($\leq .2$) in all prompt combinations against an agent that only defects once.

B Opponent strategies

B.1 Preliminaries

For the Prisoner's Dilemma and Battle of the Sexes games, we let LLMs play against simple, hand-coded strategies to further understand their behaviour. These simple strategies are designed to assess how LLMs behave when playing with more human-like players. Table 1 summarises the simple strategies we used.

Supplementary Table 1. Descriptions of hard-coded simple and human like strategies used as opponents in Prisoner's Dilemma and Battle of the Sexes games.

Game	Strategy	Description
Prisoner's Dilemma	Always defect	Defects in all rounds
	Always cooperate	Cooperates in all rounds
	False defector	Defects in the first round, then cooperates in all subsequent rounds
Battle of the Sexes	Always ballet	Chooses the ballet option in all rounds
	Always football	Chooses the football option in all rounds
	Alternate	Alternates between both options in each round, starting with the option the opponent prefers

B.2 Tit-for-tats

We investigated GPT-4's behaviour against various more common strategies including Tit-for-Tat (TFT) and its variations such as Tit-for-Two-Tats (TFTT), Suspicious Tit-for-Tat (STFT), Reverse Tit-for-Tat (RTFT), Hard Tit-for-Tat (HTFT) and Naive Prober (NP) over a longer horizon of 20 rounds.

Supplementary Table 2. Descriptions of common Tit for Tat (TFT) strategies and the Grim Trigger (GT) strategy for the iterative Prisoner's Dilemma.

Strategy	Description
Tit for Tat (TFT)	Cooperate on the first move, then copy the opponent's last move
Tit for Two Tats (TFTT)	Cooperate on the first move, and defect only when the opponent defects twice
Suspicious Tit for Tat (STFT)	Same as TFT but defect on the first move
Reverse Tit for Tat (RTFT)	Defect on the first move, then play the reverse of the opponent's last move
Hard Tit for Tat (HTFT)	Cooperate on the first move, and defect if the opponent defected on any of the previous 3 moves, else cooperate
Naive Prober 0.1 (NP10)	Like TFT, but occasionally defect with a small probability (10%)
Naive Prober 0.2 (NP20)	Like TFT, but occasionally defect with a small probability (20%)
Grim Trigger (GT)	Cooperate as long as the opponent does not defect, always defect if the opponent defects

Table 2 summarises all of the additional strategies we have investigated. We found that GPT-4 always cooperates against TFT and TFTT as these strategies start with cooperate and never defect unless the opponent defects, never triggering a defect from GPT-4. Against STFT and RTFT which both start with a defect, GPT-4 starts with cooperate and then alternates between both options only cooperating around 50% of the rounds against these opponent strategies. Against HTFT, it only cooperates in the first round and always defects in rest of the game, following the Grim Trigger strategy and also triggering an always defecting behaviour from the opponent. Against NP strategies, it cooperates until the first time it gets defected. Depending on how far along in the game this occurs, it can try cooperating again by alternating between options. To sum up, these results

highlight the tendency in GPT-4 behaviour for retaliation against being defected and the low probability of cooperation and exploration of strategies other than Grim Trigger after a single negative encounter.

C Final round behaviour

Previous studies with human players have shown that in the final round of the Prisoner's Dilemma, human players often change their behaviour compared to earlier rounds, especially when they know it is the last interaction. This phenomenon typically results in an increase in defections[1,2]. To show if this is observed in our experiments, we expanded our analysis of the Prisoner's Dilemma and Battle of the Sexes to focus on the last rounds.

Firstly, we have analysed GPT-4's behaviour in the last rounds of Prisoner's Dilemma (PD) and Battle of the Sexes (BoS) games to see if it behaves differently if it knows that is the last interaction of the game. For this, we used GPT-4's interactions between itself and other models as well as the simple strategies described in Table 1. In Bos the SCoT prompted version of GPT-4 was also included in this list of opponents. In PD, we compared the differences in the distribution of defect and cooperate in the last round round compared to the rest of the game and the effect was not significant ($\chi^2(1, N = 160) = 0.29, P = 0.592$). We also compared the frequency of answer flips between the 9th and 10th rounds compared to the rest of the game and this effect was also not significant ($\chi^2(1, N = 160) = 0.70, P = 0.401$). The same analysis in BoS revealed and again found no statistically significant difference in the distribution of both options ($\chi^2(1, N = 180) = 0.02, P = 0.880$) and the frequency of answer flips in the last round ($\chi^2(1, N = 180) = 0.35, P = 0.552$).

C.1 Games with varying ending probabilities

Next, we have added several versions of the PD game with different probabilities of ending. In these versions, GPT-4 is not informed in advance that the game will last exactly 10 rounds. Instead, GPT-4 is given the probability of the game ending after each round. We selected the probability values by calculating the threshold at which the dominant strategy shifts from cooperation to defection:

Given the probability of the game ending at any moment is Θ, the expected payoff for cooperation C can be calculated as

$$\prod^{C} = R + R(1-\Theta) + R(1-\Theta)^2 + \ldots = \frac{R}{\Theta} \tag{4}$$

in the same way, we calculate the expected payoff for defecting D as below.

$$\prod^{D} = T + P(1-\Theta) + P(1-\Theta)^2 + \ldots = T + P\left(\frac{1-\Theta}{\Theta}\right) \tag{5}$$

Additionally, cooperation can be sustained against even the grim strategies if the expected payoff for cooperation is greater than or equal to the expected payoff for defecting

$$\prod^{C} \geq \prod^{D} \tag{6}$$

When we enter the actual payoff values we use $R = 8, P = 5, T = 10, S = 0$ into these equations and solve for the equality 6, we end up with

$$\Theta \leq 60\% \tag{7}$$

meaning if the probability of the game is ending is less than or equal to 60% or the probability of the game is continuing is greater than 40% then the dominant strategy would be to cooperate.

Using these findings, we implement different versions of repeated Prisoner's Dilemma by setting the probabilities of the games ending after each round to 40%, 60% and 80%. We let GPT-4 play in these conditions against the simple strategies we summarise in Table 2 and analyse its behaviour in these more realistic scenarios suggested by the reviewer.

To do this, we changed the instructions in the prompts to include the indefinite nature of the game and the exact probability values for the continuation of the game from the old finitely repeated version of

```
You are playing a game repeatedly with another player.  In this game, you can choose
between Option J and Option F. You will play 10 rounds in total with the same player...
```

to the new version where $X = 40, 60$ or 80 as follows

```
You are playing a game repeatedly with another player.  In this game, you can choose
between Option J and Option F. You will play an indefinite number of rounds in total
with the same player.  There is a X% probability that the game will end after each round...
```

The number of rounds were still kept fixed for better reproducibility. The simulations ran for a longer (but fixed) horizon of 20 rounds.

Initial results with these 3 probabilities around the threshold of 60% showed that even a relatively small probability that a game can end at any point makes GPT-4 defect in every round. To further prove this strong tendency, we ran another version where we tell the model that the possibility of a game ending after each round is at 1%. This confirmed that GPT-4 any probability that a game may end makes GPT-4 always defect and the value of this probability has no significance in the power of the observed behaviour. We also tried a version of the prompt to include the probability of the game continuing instead of ending:

```
You are playing a game repeatedly with another player.  In this game, you can choose
between Option J and Option F. You will play an indefinite number of rounds in total
with the same player.  There is a (100−X)% probability that the game will continue after
each round...
```

and observed no change to the effect. In repeated games with probabilistic continuation, human players typically adopt a strategy that favors cooperation if the payoff from cooperation and probability of future interactions are high enough[3]. This approach is often rooted in a forward-looking perspective[4], where players weigh the long-term benefits of cooperating over immediate gains from defection. Previous studies have also shown that human players often anticipate end-game outcomes and make decisions accordingly[5–7]. On the other hand, LLMs primarily respond based on immediate context. Being trained to predict contextually plausible responses in the short term rather than to strategize for cumulative gains over a series of interactions[8], LLMs lack an inherent mechanism to prioritize future payoffs. This short-term focus may make LLMs default to defection when there is uncertainty about continuation. Without an explicit mechanism to calculate or represent expected future rewards, LLMs may prioritize immediate gains of defection over cooperative behaviour that could bring higher long-term payoffs.

Additionally, recent work demonstrates how LLMs struggle with bidirectional reasoning tasks[9]. This limitation suggests that inverting reasoning from final-game scenarios backward through earlier rounds is challenging for LLMs. Furthermore, LLMs trained predominantly on unidirectional sequences may not inherently grasp reverse-causal structures which are also fundamental to backward induction in repeated games. When playing the final round in repeated games, LLMs might default to their standard response patterns rather than adjusting based on the move's impact, as humans typically do. This has also been limiting in tasks requiring multi-step reasoning, where LLMs need additional structural prompting to sustain complex dependencies across a sequence[10].

D Human participant study

Online interface. The participants' interface (Fig. 4) was designed to provide clear and actionable information about the current game. It consisted of three key components: a payoff matrix displaying the outcomes of each option, a history panel showing past choices and points for both players, and an indicator of the remaining rounds alongside the two available options and corresponding keys.

Prompts and human instructions. The cover story used for interactions with both LLMs and human participants was content-wise identical to ensure consistent framing. However, the presentation was adapted to suit each audience. For human participants, visual cues and concise text were prioritized to create a more engaging experience. For example, while payoff matrices were presented as text for the LLMs (see Appendix A for the detailed prompt progression), humans were shown a visual matrix (Fig. 4a). Similarly, the game history was detailed in textual form for the LLMs but summarized in a table for human participants (Fig. 4b), containing all relevant information.

Ananlysis of Participants' Temporal Behaviour Within the Games

We additionally investigated participants' temporal behavior in both games. For Battle of the Sexes, participants correlated their responses with their previous actions ($r = 0.32$, $t = 14.15$, $p < .001$, $BF > 100$) as well as with the previous actions of the agents ($r = 0.16$, $t = 5.52$, $p < .001$, $BF > 100$). Moreover, a successful coordination on the previous trial was predictive of a successful coordination on the next trial ($r = 0.13$, $t = 5.71$, $p < .001$, $BF > 100$). We found in the main analysis that the amount of cooperation increased for games with agents using social chain-of-thought prompting. Thus, we wanted to analyze this pattern further to investigate what drove this increase. For this, we first analyzed participants' cycling behavior. We defined

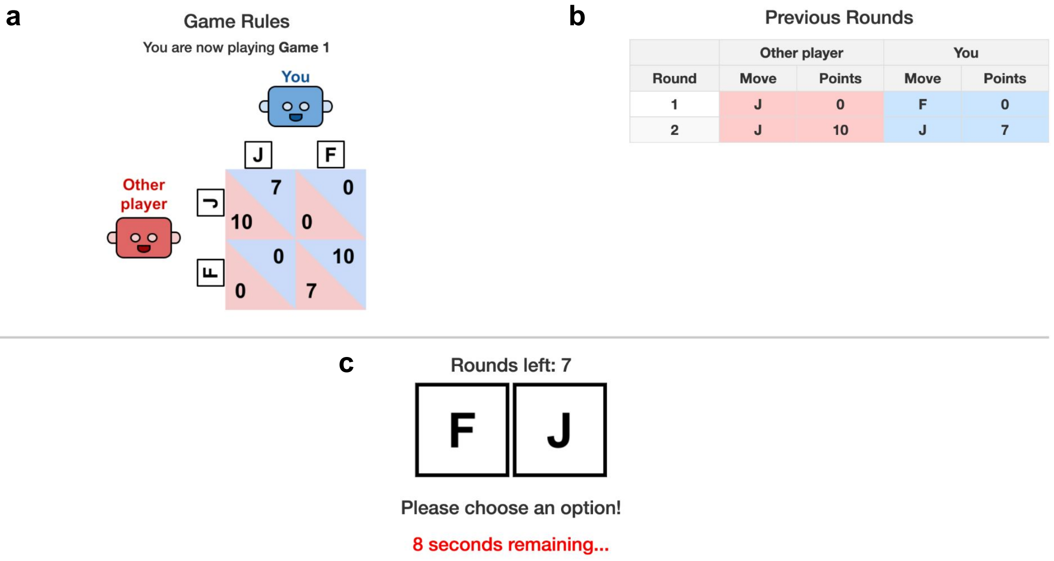

Supplementary Figure 4. Participant interface in the online study. The interface consists of three components: **a:** a matrix summarizing the payoffs for each option, **b:** the game history showing the options chosen and points received by both players in previous rounds, and **c:** the number of remaining rounds in the current game, along with the two available options and the keys to press to select them.

a complete cycle as participants either sequentially choosing Football-Ballet-Football-Ballet or Ballet-Football-Ballet-Football. We found that ca. 25% of participants exhibited such cycling behavior, while there was no difference as in to which agents they played with ($t(191 = 1.73$, $p = .08$, $BF = 0.62$). What was different, however, between the two groups was that the social chain-of-thought prompted agents chose their own preferred options significantly less often ($t(191) = 4.53$, $p < .001$ $BF = 7.4$), $BF = 3.34$). Thus, the improvement in successful cooperation behaviors in the Battle of the Sexes mostly resulted from agents rather than participants changing their behavior.

For the Prisoner's Dilemma, participants also correlated their responses with their previous actions ($r = 0.52$, $t = 25.24$, $p < .001$, $BF > 100$) as well as with the previous actions of the agents ($r = 0.51$, $t = 25.21$, $p < .001$, $BF > 100$). Additionally, we assessed participants' overall strategies by implementing three models: grim trigger, tit-for-tat, and forgiving tit-for-tat. The grim trigger strategy starts out cooperating but exclusively defects after the first encountered defection. Tit-for-tat starts our by cooperating and plays on the current round whatever the other player played on the last round. Forgiving tit-for-tat starts out by cooperating and –while mirroring the other player's action from the last round– only defects if the other agent defected more than once. We assess how often these strategies correctly predict participants' actions. We find that the grim trigger model predicts 91 participants best, tit-for-tat predicts 18 participants best, and forgiving tit-for-tat predicts 86 participants best. While we find now significant relationship between the type of opponent and how well either the grim trigger ($r = 0.08$, $t = 1.63$ $p = .10$, $BF = 0.6$) or tit-for-tat predicts participants choices ($r = 0.09$, $t = 2.04$, $p = .04$, $BF = 1.22$), participants seem to be significantly better predicted by the forgiving tit-for-tat strategy in the social chain-of-thought condition ($r = 0.16$, $t = 3.91$, $p = .001$, $BF = 7.3$). Thus, participants change their behavior when playing with the social chain-of-thought prompted model by becoming themselves more forgiving.

E Discussion

We believe that understanding LLM behaviour through the lens of game theory is particularly crucial to understand these systems' long-term societal implications. This is because — despite their seeming simplicity — the games we investigate capture key issues that humans face: For example, coordination games like the Battle of the Sexes have been used to describe societal phenomena like the concentration of industries or gentrification[11]. Additionally, selfish behaviour in the prisoner's dilemma akin to what we observe is often taken as a model of (the failure of) collaboration on climate change[12]. As LLMs keep proliferating and gaining increasing decision-making autonomy, it is thus crucial to understand how their social behaviour unfolds, from well-controlled experimental environments to decisions in the wild. Our behavioural game theory for machines is a first step in this direction.

References

1. Selten, R. & Stoecker, R. End behavior in sequences of finite prisoner's dilemma supergames a learning theory approach. *J. Econ. Behav. & Organ.* **7**, 47–70 (1986).
2. Kreps, D. M., Milgrom, P., Roberts, J. & Wilson, R. Rational cooperation in the finitely repeated prisoners' dilemma. *J. Econ. theory* **27**, 245–252 (1982).
3. Dal Bó, P. & Fréchette, G. R. The evolution of cooperation in infinitely repeated games: Experimental evidence. *Am. Econ. Rev.* **101**, 411–429 (2011).
4. Nowak, M. A. & Sigmund, K. Evolution of indirect reciprocity. *Nature* **437**, 1291–1298 (2005).
5. Axelrod, R. & Hamilton, W. D. The evolution of cooperation. *science* **211**, 1390–1396 (1981).
6. Nowak, M. A. Five rules for the evolution of cooperation. *science* **314**, 1560–1563 (2006).
7. Dreber, A., Fudenberg, D. & Rand, D. G. Who cooperates in repeated games: The role of altruism, inequity aversion, and demographics. *J. Econ. Behav. & Organ.* **98**, 41–55 (2014).
8. Radford, A. *et al.* Language models are unsupervised multitask learners. *OpenAI blog* **1**, 9 (2019).
9. Berglund, L. *et al.* The reversal curse: Llms trained on" a is b" fail to learn" b is a". *arXiv preprint arXiv:2309.12288* (2023).
10. Wei, J. *et al.* Chain-of-thought prompting elicits reasoning in large language models. *Adv. neural information processing systems* **35**, 24824–24837 (2022).
11. Camerer, C. F. *Behavioral game theory: Experiments in strategic interaction* (Princeton university press, 2011).
12. Wood, P. J. Climate change and game theory. *Annals New York Acad. Sci.* **1219**, 153–170 (2011).
