# Supplementary material for: Playing repeated games with large language models
Source: Nat Hum Behav. 2025 May 8;9(7):1380–90. doi: 10.1038/s41562-025-02172-y (PMC12283376; doi:10.1038/s41562-025-02172-y)
Supplement: Supplementary file 2 — Reporting Summary [file 41562_2025_2172_MOESM2_ESM.pdf]

nature portfolio

Corresponding author(s): Elif Akata

Last updated by author(s): Feb 25, 2025

Reporting Summary

Nature Portfolio wishes to improve the reproducibility of the work that we publish. This form provides structure for consistency and transparency in reporting. For further information on Nature Portfolio policies, see our Editorial Policies and the Editorial Policy Checklist.

Statistics

For all statistical analyses, confirm that the following items are present in the figure legend, table legend, main text, or Methods section.

n/a	Confirmed	
☐	☒	The exact sample size (*n*) for each experimental group/condition, given as a discrete number and unit of measurement
☐	☒	A statement on whether measurements were taken from distinct samples or whether the same sample was measured repeatedly
☐	☒	The statistical test(s) used AND whether they are one- or two-sided<br>*Only common tests should be described solely by name; describe more complex techniques in the Methods section.*
☒	☐	A description of all covariates tested
☒	☐	A description of any assumptions or corrections, such as tests of normality and adjustment for multiple comparisons
☐	☒	A full description of the statistical parameters including central tendency (e.g. means) or other basic estimates (e.g. regression coefficient) AND variation (e.g. standard deviation) or associated estimates of uncertainty (e.g. confidence intervals)
☐	☒	For null hypothesis testing, the test statistic (e.g. *F*, *t*, *r*) with confidence intervals, effect sizes, degrees of freedom and *P* value noted<br>*Give P values as exact values whenever suitable.*
☐	☒	For Bayesian analysis, information on the choice of priors and Markov chain Monte Carlo settings
☒	☐	For hierarchical and complex designs, identification of the appropriate level for tests and full reporting of outcomes
☐	☒	Estimates of effect sizes (e.g. Cohen's *d*, Pearson's *r*), indicating how they were calculated

Our web collection on statistics for biologists contains articles on many of the points above.

Software and code

Policy information about availability of computer code

Data collection	For our model tasks, we used the public OpenAI API with the GPT-4, text-davinci-003 and text-davinci-002 models which are available via the completion endpoint, Meta AI's Llama 2 70B Chat model, and the Anthropic API model Claude 2 to run our simulations. Human data was collected via Prolific using an online experiment written in JavaScript/HTML/CSS.
Data analysis	Behavioural data was analysed using R (4.4.0) and Python (3.11). The model simulations were implemented in Python. The code underlying this study, prompt variations and model simulations are available on github.com/eliaka/repeatedgames.

For manuscripts utilizing custom algorithms or software that are central to the research but not yet described in published literature, software must be made available to editors and reviewers. We strongly encourage code deposition in a community repository (e.g. GitHub). See the Nature Portfolio guidelines for submitting code & software for further information.

Data

Policy information about availability of data

All manuscripts must include a data availability statement. This statement should provide the following information, where applicable:

- Accession codes, unique identifiers, or web links for publicly available datasets
- A description of any restrictions on data availability
- For clinical datasets or third party data, please ensure that the statement adheres to our policy

All participant and model simulation data from the experiments are publicly available on GitHub (https://github.com/eliaka/repeatedgames).

Human research participants

Policy information about studies involving human research participants and Sex and Gender in Research.

Reporting on sex and gender	Sex and gender were not relevant in study design. The study was distributed evenly to male and female participants. Sex and gender was determined based on participant demographics on the Prolific platform.
Population characteristics	N=195, 89 females, mean age=26.72, SD=4.19.
Recruitment	Participants were recruited from Prolific and were required to be fluent speakers of English. The study may exhibit self-selection bias since participants voluntarily joined via Prolific and were limited to fluent English speakers, potentially reducing generalisability to non-English speaking populations. However, the behavioral games used are based on universal decision-making principles. The use of English speakers was due to language proficiency, not a theoretical limitation.
Ethics oversight	Ethics committee of the Eberhard Karls University Tübingen (protocol nr. 701/2080BO)

Note that full information on the approval of the study protocol must also be provided in the manuscript.

Field-specific reporting

Please select the one below that is the best fit for your research. If you are not sure, read the appropriate sections before making your selection.

☐ Life sciences ☒ Behavioural & social sciences ☐ Ecological, evolutionary & environmental sciences

For a reference copy of the document with all sections, see nature.com/documents/nr-reporting-summary-flat.pdf

Behavioural & social sciences study design

All studies must disclose on these points even when the disclosure is negative.

Study description	Quantitative study. We let large language models play finitely repeated games with each other, with human-like strategies and human players using two-player, two-action games from behavioural economics.
Research sample	Our sample consisted of participants (N=195, mean age=26.72, SD=4.19.) recruited via Prolific, with gender counterbalanced, all required to be fluent in English. While not fully representative of the general population due to self-selection and language criteria, this sample was chosen to ensure clear communication and reliable engagement with the behavioral game theory tasks. Additionally, the study featured five language models: OpenAI's GPT-4, text-davinci-003, text-davinci-002, Meta AI's Llama 2 70B Chat, and Anthropic's Claude 2. All models played 2x2 games against each other and hand-coded human-like strategies, with GPT-4 acting as the LLM opponent in the human participant study.
Sampling strategy	Participants were recruited via Prolific using a convenience sampling approach, with stratification to ensure gender counterbalancing. The sample size of N=195 was chosen based on comparable behavioral game theory studies in the literature. Participants played both the Prisoner's Dilemma and the Battle of the Sexes, with the order counterbalanced between subjects. Models were chosen so that they adequately represent current SOTA models (large and small, open source and closed source).
Data collection	The open-source models were evaluated on a Slurm-based cluster with a single A100. For proprietary models, we used the public APIs. Human participant data was collected on Prolific. Data collection code is available on GitHub (github.com/eliaka/repeatedgames). The researcher was not blinded to experimental conditions. In the model experiments, the language models played the games autonomously. In the human study, the design did not require reciprocal interaction with the researcher and it compared two model conditions (prompted vs. baseline). All participants were fully debriefed after the experiment.
Timing	The experiments on Prolific took place between 17/06/2024 and 21/06/2024.
Data exclusions	We excluded data of 21 players who failed to make a round's choice between the 2 options within a given time frame (20 seconds).
Non-participation	14 participants did not complete the study; two of them having internet connection issues, one quoting technical problems and the rest without explicit reasoning.
Randomization	Participants were randomly allocated into groups.

Reporting for specific materials, systems and methods

We require information from authors about some types of materials, experimental systems and methods used in many studies. Here, indicate whether each material, system or method listed is relevant to your study. If you are not sure if a list item applies to your research, read the appropriate section before selecting a response.

Materials & experimental systems

n/a	Involved in the study
☒	☐ Antibodies
☒	☐ Eukaryotic cell lines
☒	☐ Palaeontology and archaeology
☒	☐ Animals and other organisms
☒	☐ Clinical data
☒	☐ Dual use research of concern

Methods

n/a	Involved in the study
☒	☐ ChIP-seq
☒	☐ Flow cytometry
☒	☐ MRI-based neuroimaging
